# Supplementary material for: Drivers and pressures behind insect decline in Central and Western Europe based on long-term monitoring data
Source: PLoS One. 2023 Aug 23;18(8):e0289565. doi: 10.1371/journal.pone.0289565 (PMC10446172; doi:10.1371/journal.pone.0289565)
Supplement: S1 Table — (PDF) [file pone.0289565.s001.pdf]

Table S1: Developed categories with lists of keywords in English and German.

Keyword category 1: taxonomic group	
Scientific name	Trivial names engl./*germ.*
Carabidae	ground beetles, carabid beetles / *Laufkäfer*
Lepidoptera	Butterflies, moths / *Schmetterlinge, Falter*
Keyword category 2: time-based/methodical context	
Monitoring, long-term study/data	*Monitoring, Langzeitdaten, Langzeitstudie*
Decline, loss, decrease, increase	*Rückgang, Verlust, Abnahme, Zunahme*
Progression, change	*Entwicklung, Veränderung, Entwicklung*
Keyword category 3: measured parameters	
Biomass, biodiversity, abundance, number of species, distribution	*Biomasse, Biodiversität, Abundanz, Artenzahl, Verbreitung*
Keyword category 4: land use/agricultural context	
agricultural landscape, field, arable land, agricultural, arable, agriculture, crop	*Agrarlandschaft, agrar, Acker, Landwirtschaft, landwirtschaftliche Nutzung, Anbaufläche*
buffer strips, wildflower strips, field margins	*Randstreifen, Ackerrandstreifen*
fallow (ground), pasture, meadow, grassland	*Brache, Ackerbrache, Weide, Wiese, Grasland*
crops (maise, cabbage, corn, rapeseed)	*Feldfrüchte (Mais, Kohl, Getreide, Raps)*
Human impact OR anthropogenic impact	*anthropogene Beeinflussung*
