# Supplementary material for: Drivers and pressures behind insect decline in Central and Western Europe based on long-term monitoring data
Source: PLoS One. 2023 Aug 23;18(8):e0289565. doi: 10.1371/journal.pone.0289565 (PMC10446172; doi:10.1371/journal.pone.0289565)
Supplement: S2 Table — (see S4 Table). (PDF) [file pone.0289565.s002.pdf]

Table S2: Description of drivers, pressure class and state categories, obtained from the evaluated literature (see S4 Table).

Category	Description
**Driver**	
agricultural extensification	Reduction of the agricultural production, characterised by e. g. a decrease in livestock, fertilisation or use of pesticides
agricultural intensification	Augmentation of the agricultural production, characterised by e. g. an increase in field size, livestock, fertilisation or use of pesticides
agriculture in general	Summarisation of all farming activities
anthropogenic activities in general	Summarisation of all human activities, e. g. agriculture, urban construction or traffic
climate change	Change of climate, mainly caused by anthropogenic emissions of greenhouse gases since the beginning of the Industrial Age
globalisation	Increase in worldwide linkages between nations, individuals or societies in different sectors, e. g. economy, culture, communication or environment
various drivers	Combinations of several, not differentiable drivers
natural drivers	Combination of different natural drivers, e. g. weather and climate in general
nature conservation	Summarisation of human activities to improve the natural environment
silvicultural intensification	Increase in silvicultural production, e. g. through the cultivation of monocultures
urbanisation	Augmentation of city areas, e. g. the construction of new buildings and transportation infrastructure, or the infrastructural development of rural regions
none	No pressures were discussed
**Pressure class**	
climate	Summarisation of different meteorological, dynamic processes in the terrestrial atmosphere, averaged over a longer time period (e. g. 30 years)
collection activity	Direct killing of animals by human beings, e. g. through entomologists collecting organisms for research purposes
eutrophication	Man-induced accumulation of nutrients in the environment
fertiliser	Application of organic or mineral fertilisers
forestation	Woodland cultivation
introduced species	Non-indigenous species that have arrived in an area outside their natural distribution by human activities
land consumption	Vanishing of an area that was formerly a habitat for a species, a population or a biocenosis, e. g. by road building or urbanisation
land use	The use of a landscape area for a definded purpose (e. g. forestation, intensive or extensive agriculture)
management practices	Detailed agri-, horti- and silvicultural practices, such as cutting regimes, grazing or liming
various pressures	Summarisation of different pressures
pesticides	Application of plant protection products (insecticides, herbicides, fungicides etc.)

pollution	Release of harmful substances as well as adverse physical factors into the environment, such as different chemicals or light and noise of anthropogenic sources
resource availability	Occurence of the resources needed by the animal groups, e. g. food and host plants
succession	Natural development of an area after a disturbance, e. g. after the ceasing or reduction of cultivation
none	No pressures were discussed
**State**	
habitat & organisms	The habitats as well as directly the organisms are affected by the pressures
habitat availability	The pressures affect the presence of specific, usable habitats
habitat condition	The pressures influence the habitat characteristics
organisms	The organisms are directly affected by the pressures
none	No pressures were discussed
