# Supplementary material for: Drivers and pressures behind insect decline in Central and Western Europe based on long-term monitoring data
Source: PLoS One. 2023 Aug 23;18(8):e0289565. doi: 10.1371/journal.pone.0289565 (PMC10446172; doi:10.1371/journal.pone.0289565)
Supplement: S4 Table — (PDF) [file pone.0289565.s004.pdf]

Table S4: Full list of analysed publications.

Authors short	Taxa	Full citation
Aebischer & Potts(1990)	Carabidae	Aebischer, N. J., & Potts, G. R. (1990). Long-term changes in numbers of cereal invertebrates assessed by monitoring. Brighton Crop Protection Conference - Pests and Diseases, 3B-4, 163–172.
Artmann-Graf(2015)	Lepidoptera	Artmann-Graf, G. (2015). Tagfalter in der zentralen Nordwestschweiz gestern und heute Verbreitungsatlas und Monitoring (1st ed.). Pro Natura Solothurn.
Asher et al(2011)	Lepidoptera	Asher, J., Fox, R., & Warren, M. S. (2011). British butterfly distributions and the 2010 target. Journal of Insect Conservation, 15(1–2), 291–299. https://doi.org/10.1007/s10841-010-9346-7
Augenstein et al(2012)	Lepidoptera, Carabidae	Augenstein, B., Ulrich, W., & Habel, J. C. (2012). Directional temporal shifts in community structure of butterflies and ground beetles in fragmented oligotrophic grasslands of Central Europe. Basic and Applied Ecology, 13(8), 715–724. https://doi.org/10.1016/j.baae.2012.09.004
Basedow(1987)	Carabidae	Basedow, T. (1987). Der Einfluß gesteigerter Bewirtschaftungsintensität im Getreidebau auf die Laufkäfer. Auswertung vierzehnjähriger Untersuchungen (1971–1984). Mitteilungen Aus Der Biologischen Bundesanstalt Für Land- Und Forstwirtschaft Berlin-Dahlem, 235.
Basedow(1998)	Carabidae	Basedow, T. (1998). Langfristige Bestandsveränderungen von Arthropoden in der Feldflur, ihre Ursachen und deren Bedeutung für den Naturschutz, gezeigt an Laufkäfern (Carabidae) in Schleswig-Holstein, 1971–96. Schriftenreihe Für Landschaftspflege Und Naturschutz, 58, 215–227.
Brereton et al(2009)	Lepidoptera	Brereton, T., van Swaay, C., & van Strien, A. (2009). Developing a butterfly indicator to assess changes in Europe's biodiversity. Conference Proceedings of the European Bird Census Council Bird, 78–97.
Brereton et al(2019)	Lepidoptera	Brereton, T., Dennis, E., Fox, R., Byron, M., Perscott, T., Shortall, C., & Foster, S. (2019). Trend note - Trends of Moths in Scotland.pdf (Trend Note No. 018). Scottisch Natural Heritage.
Brooks et al(2012)	Carabidae	Brooks, D. R., Bater, J. E., Clark, S. J., Monteith, D. T., Andrews, C., Corbett, S. J., Beaumont, D. A., & Chapman, J. W. (2012). Large carabid beetle declines in a United Kingdom monitoring network increases evidence for a widespread loss in insect biodiversity. Journal of Applied Ecology, 49(5), 1009–1019. https://doi.org/10.1111/j.1365-2664.2012.02194.x
Conrad et al(2004)	Lepidoptera	Conrad, K. F., Woiwod, I. P., Parsons, M., Fox, R., & Warren, M. S. (2004). Long-term population trends in widespread British moths. Journal of Insect Conservation, 8(2–3), 119–136.
Conrad et al(2006)	Lepidoptera	Conrad, K. F., Warren, M. S., Fox, R., Parsons, M. S., & Woiwod, I. P. (2006). Rapid declines of common, widespread British moths provide evidence of an insect biodiversity crisis. Biological Conservation, 132(3), 279–291. https://doi.org/10.1016/j.biocon.2006.04.020
Coulthard et al(2019)	Lepidoptera	Coulthard, E., Norrey, J., Shortall, C., & Harris, W. E. (2019). Ecological traits predict population changes in moths. Biological Conservation, 233, 213–219. https://doi.org/10.1016/j.biocon.2019.02.023
Den Boer & Van Dijk(1994)	Carabidae	Den Boer, P. J., & Van Dijk, T. S. (1994). Carabid beetles in a changing environment. Wageningen Agricultural University Papers, 94(6), 30.
Dennis et al(2019)	Lepidoptera	Dennis, E. B., Brereton, T. M., Morgan, B. J. T., Fox, R., Shortall, C. R., Prescott, T., & Foster, S. (2019). Trends and indicators for quantifying moth abundance and occupancy in Scotland. Journal of Insect Conservation, 23(2), 369–380. https://doi.org/10.1007/s10841-019-00135-z

Desender & Turin(1989)	Carabidae	Desender, K., & Turin, H. (1989). Loss of habitats and changes in the composition of the ground and tiger beetle fauna in four West European countries since 1950 (Coleoptera: Carabidae, Cicindelidae). Biological Conservation, 48(4), 277–294.
Desender et al(1994)	Carabidae	Desender, K., Dufrêne, M., & Maelfait, J.-P. (1994). Long term dynamics of carabid beetles in Belgium: A preliminary analysis on the influence of changing climate and land use by means of a database covering more than a century. In Carabid beetles: Ecology and evolution (pp. 247–252). Springer.
Desender et al(2010)	Carabidae	Desender, K., Dekoninck, W., Dufrêne, M., & Maes, D. (2010). Changes in the distribution of carabid beetles in Belgium revisited: Have we halted the diversity loss? Biological Conservation, 143(6), 1549–1557. https://doi.org/10.1016/j.biocon.2010.03.039
Desender(1986)	Carabidae	Desender, K. (1986). Distribution and ecology of carabid beetles in Belgium (Coleoptera, Carabidae) Part 1-4. Institut Royal des Sciences Naturelles de Belgique.
Ewald et al(2015)	Carabidae	Ewald, J. A., Wheatley, C. J., Aebischer, N. J., Moreby, S. J., Duffield, S. J., Crick, H. Q. P., & Morecroft, M. B. (2015). Influences of extreme weather, climate and pesticide use on invertebrates in cereal fields over 42 years. Global Change Biology, 21(11), 3931–3950. https://doi.org/10.1111/gcb.13026
Eyre et al(2016)	Carabidae	Eyre, M. D., Sanderson, R. A., McMillan, S. D., & Critchley, C. N. R. (2016). Crop cover the principal influence on non-crop ground beetle (Coleoptera, Carabidae) activity and assemblages at the farm scale in a long-term assessment. Bulletin of Entomological Research, 106(2), 242–248. https://doi.org/10.1017/S0007485315001054
Fartmann(2004)	Lepidoptera	Fartmann, T. (2004). Die Schmetterlingsgemeinschaften der Halbtrockenrasen-Komplexe des Diemeltales—Biozönologie von Tagfaltern und Widderchen in einer alten Hudelandschaft. Abhandlungen aus dem Westfälischen Museum für Naturkunde, 66(1), 273.
Fox et al(2014)	Lepidoptera	Fox, R., Brereton, T. M., Asher, J., August, T. A., Botham, M. S., Bourn, N. A. D., Cruickshanks, K. L., Bulman, C. R., Ellis, S., & Harrower, C. A. (2015). The State of the UK's Butterflies 2015.
Fox et al(2015)	Lepidoptera	Fox, Richard, Oliver, T. H., Harrower, C., Parsons, M. S., Thomas, C. D., & Roy, D. B. (2014). Long-term changes to the frequency of occurrence of British moths are consistent with opposing and synergistic effects of climate and land-use changes. Journal of Applied Ecology, 51(4), 949–957. https://doi.org/10.1111/1365-2664.12256
Gonzalez-Megias et al(2008)	Lepidoptera	González-Megías, A., Menéndez, R., Roy, D., Brereton, T., & Thomas, C. D. (2008). Changes in the composition of British butterfly assemblages over two decades. Global Change Biology, 14(7), 1464–1474. https://doi.org/10.1111/j.1365-2486.2008.01592.x
Gottschalk & Komrowski(2017)	Lepidoptera	Gottschalk, T. K., & Komrowski, A. (2017). Landnutzungsveränderungen am Spitzberg bei Tübingen. Naturschutz und Landschaftsplanung, 13.
Groenendijk & Ellis(2010)	Lepidoptera	Groenendijk, D., & Ellis, W. N. (2011). The state of the Dutch larger moth fauna. Journal of Insect Conservation, 15(1–2), 95–101. https://doi.org/10.1007/s10841-010-9326-y
Groenendijk & Van der Meulen(2004)	Lepidoptera	Groenendijk, Dick, & van der Meulen, J. (2004). Conservation of moths in The Netherlands: Population trends, distribution patterns and monitoring techniques of day-flying moths. Journal of Insect Conservation, 8(2–3), 109–118.
Günther & Assmann(2004)	Carabidae	Günther, J., & Assmann, T. (2004). Fluctuations of carabid populations inhabiting an ancient woodland (Coleoptera, Carabidae). Pedobiologia, 48(2), 159–164.
Habel et al(2016)	Lepidoptera	Habel, J. C., Segerer, A., Ulrich, W., Torchyk, O., Weisser, W. W., & Schmitt, T. (2016). Butterfly community shifts over two centuries:

		Shifts in Butterfly Communities. Conservation Biology, 30(4), 754–762. https://doi.org/10.1111/cobi.12656
Habel et al(2019a)	Lepidoptera	Habel, J. C., Segerer, A. H., Ulrich, W., & Schmitt, T. (2019). Succession matters: Community shifts in moths over three decades increases multifunctionality in intermediate successional stages. Scientific Reports, 9(1), 5586. https://doi.org/10.1038/s41598-019-41571-w
Habel et al(2019b)	Lepidoptera	Habel, J. C., Trusch, R., Schmitt, T., Ochse, M., & Ulrich, W. (2019). Long-term large-scale decline in relative abundances of butterfly and burnet moth species across south-western Germany. Scientific Reports, 9(1), 14921. https://doi.org/10.1038/s41598-019-51424-1
Hacker(1995)	Lepidoptera	Hacker, H. (1995). Bestandsentwicklung und-rückgang einheimischer Schmetterlinge in diesem Jahrhundert, dargestellt am Beispiel des Landkreises Lichtenfels (nördlichster Frankenjura). Beiträge Zur Bayerischen Entomofaunistik, 1, 97.
Hallmann et al(2019)	Lepidoptera, Carabidae	Hallmann, C. A., Zeegers, T., Klink, R., Vermeulen, R., Wielink, P., Spijkers, H., Deijk, J., Steenis, W., & Jongejans, E. (2020). Declining abundance of beetles, moths and caddisflies in the Netherlands. Insect Conservation and Diversity, 13(2), 127–139. https://doi.org/10.1111/icad.12377
Hanisch(2009)	Lepidoptera	Hanisch, K. (2009). Tagfalter im Gebiet der Stadt Köln einschließlich Königsforst und Wahner Heide – ehemals und heute (Lep., Hesperioidea et Papilionidea). Melanargia, 21(4), 137–226.
Herrig et al(2014)	Lepidoptera	Herrig, A., Schmitt, T., Weitzel, M., & Filz, K. (2014). Zusammensetzung und Bestandsentwicklung von Tagfalterzönosen (Lepidoptera: Rhopalocera) auf Brachflächen im Raum Trier über 40 Jahre. Mainzer Naturwissenschaftliches Archiv, 51, 357–392.
Heydemann(1983)	Carabidae	Heydemann, B. (1983). Aufbau von Ökosystemen im Agrarbereich und ihre langfristigen Veränderungen. Daten Und Dokumente Zum Umweltschutz, 35, 53–84.
Homburg et al(2019)	Carabidae	Homburg, K., Drees, C., Boutaud, E., Nolte, D., Schuett, W., Zumstein, P., Ruschkowski, E., & Assmann, T. (2019). Where have all the beetles gone? Long-term study reveals carabid species decline in a nature reserve in Northern Germany. Insect Conservation and Diversity, icad.12348. https://doi.org/10.1111/icad.12348
Irmler(2003)	Carabidae	Irmler, U. (2003). The spatial and temporal pattern of carabid beetles on arable fields in northern Germany (Schleswig-Holstein) and their value as ecological indicators. Agriculture, Ecosystems & Environment, 98(1–3), 141–151. https://doi.org/10.1016/S0167-8809(03)00076-8
Irmler(2018)	Carabidae	Irmler, U. (2018). Which carabid species (Coleoptera: Carabidae) profit from organic farming after a succession of 15 years? Agriculture, Ecosystems & Environment, 263, 1–6. https://doi.org/10.1016/j.agee.2018.02.019
Irmler(2019)	Carabidae	Irmler, U. (2019). Conversion from conventional to organic land-use on Ritzerau Manor – The winter-active ground beetles –. Angewandte Carabidologie, 13, 25–35.
Kinsella et al(2019)	Lepidoptera	Kinsella, R. S., Thomas, C. D., Crawford, T. J., Hill, J. K., Mayhew, P. J., & Macgregor, C. J. (2019). Unlocking the potential of historical abundance datasets to study biomass change in flying insects [Preprint]. Ecology. https://doi.org/10.1101/695635
Laussmann et al(2010)	Lepidoptera	Laussmann, T., Radtke, A., Wiemert, T., & Dahl, A. (2010). 150 Jahre Schmetterlingsbeobachtung in Wuppertal – Auswirkungen von Klima- und Landschaftsveränderungen (Lepidoptera). Entomologische Zeitschrift, 9.
Lenz & Schulten(2005)	Lepidoptera	Lenz, N., & Schulten, D. (2005). Tagfalter (Lep., Hesperioidea et Papilionoidea) im Gebiet der Landeshauptstadt Düsseldorf um 1900 und um 2000–ein Beispiel für alarmierende Artenverarmung im 20. Jahrhundert. Melanargia, 17(I), 19–29.

Lütolf et al(2009)	Lepidoptera	Lütolf, M., Guisan, A., & Kienast, F. (2009). History Matters: Relating Land-Use Change to Butterfly Species Occurrence. Environmental Management, 43(3), 436–446. https://doi.org/10.1007/s00267-008-9256-6
Macgregor et al(2019)	Lepidoptera	Macgregor, C. J., Williams, J. H., Bell, J. R., & Thomas, C. D. (2019). Moth biomass increases and decreases over 50 years in Britain. Nature Ecology & Evolution, 3(12), 1645–1649. https://doi.org/10.1038/s41559-019-1028-6
MacLeod et al(2004)	Carabidae	MacLeod, A., Wratten, S. D., Sotherton, N. W., & Thomas, M. B. (2004). 'Beetle banks' as refuges for beneficial arthropods in farmland: Long-term changes in predator communities and habitat. Agricultural and Forest Entomology, 6(2), 147–154. https://doi.org/10.1111/j.1461-9563.2004.00215.x
Maes & Van Dyck(2001)	Lepidoptera	Maes, D., & Van Dyck, H. (2001). Butterfly diversity loss in Flanders (north Belgium): Europe's worst case scenario? Biological Conservation, 99(3), 263–276.
Maes et al(2012)	Lepidoptera	Maes, D., Vanreusel, W., Jacobs, I., Berwaerts, K., & Van Dyck, H. (2012). Applying IUCN Red List criteria at a small regional level: A test case with butterflies in Flanders (north Belgium). Biological Conservation, 145(1), 258–266. https://doi.org/10.1016/j.biocon.2011.11.021
Morecroft et al(2009)	Lepidoptera, Carabidae	Morecroft, M. D., Bealey, C. E., Beaumont, D. A., Benham, S., Brooks, D. R., Burt, T. P., Critchley, C. N. R., Dick, J., Littlewood, N. A., Monteith, D. T., Scott, W. A., Smith, R. I., Walmsley, C., & Watson, H. (2009). The UK Environmental Change Network: Emerging trends in the composition of plant and animal communities and the physical environment. Biological Conservation, 142(12), 2814–2832. https://doi.org/10.1016/j.biocon.2009.07.004
Outhwaite et al(2020)	Carabidae	Outhwaite, C. L., Gregory, R. D., Chandler, R. E., Collen, B., & Isaac, N. J. B. (2020). Complex long-term biodiversity change among invertebrates, bryophytes and lichens. Nature Ecology & Evolution, 4(3), 384–392. https://doi.org/10.1038/s41559-020-1111-z
Paehler et al(2019)	Lepidoptera	Pähler, R., Dudler, H., & Hille, A. (2019). Das stille Sterben der Schmetterlinge / The silent demise of butterflies and moths. Eigenverlag.
Platts et al(2019)	Lepidoptera, Carabidae	Platts, P. J., Mason, S. C., Palmer, G., Hill, J. K., Oliver, T. H., Powney, G. D., Fox, R., & Thomas, C. D. (2019). Habitat availability explains variation in climate-driven range shifts across multiple taxonomic groups. Scientific Reports, 9(1), 15039. https://doi.org/10.1038/s41598-019-51582-2
Pollard et al(1995)	Lepidoptera	Pollard, E., Moss, D., & Yates, T. J. (1995). Population Trends of Common British Butterflies at Monitored Sites. The Journal of Applied Ecology, 32(1), 9. https://doi.org/10.2307/2404411
Pollard et al(1998)	Lepidoptera	Pollard, E., Woiwod, I. P., Greatorex-Davies, J. N., Yates, T. J., & Welch, R. C. (1998). The spread of coarse grasses and changes in numbers of lepidoptera in a woodland nature reserve. Biological Conservation, 84(1), 17–24. https://doi.org/10.1016/S0006-3207(97)00090-6
Pollard(1982)	Lepidoptera	Pollard, E. (1982). Monitoring butterfly abundance in relation to the management of a nature reserve. Biological Conservation, 24(4), 317–328. https://doi.org/10.1016/0006-3207(82)90018-0
Pollard(1991)	Lepidoptera	Pollard, Ernest. (1991). Monitoring butterfly numbers. In Monitoring for conservation and ecology (pp. 87–111). Springer.
Pozsgai & Littlewood(2014)	Carabidae	Pozsgai, G., & Littlewood, N. A. (2014). Ground beetle (Coleoptera: Carabidae) population declines and phenological changes: Is there a connection? Ecological Indicators, 41, 15–24.
Pozsgai et al(2016)	Carabidae	Pozsgai, G., Baird, J., Littlewood, N. A., Pakeman, R. J., & Young, M. R. (2016). Long-term changes in ground beetle (Coleoptera: Carabidae) assemblages in Scotland. Ecological Entomology, 41(2), 157–167.

Rada et al(2019)	Lepidoptera	Rada, S., Schweiger, O., Harpke, A., Kühn, E., Kuras, T., Settele, J., & Musche, M. (2019). Protected areas do not mitigate biodiversity declines: A case study on butterflies. Diversity and Distributions, 25(2), 217–224. https://doi.org/10.1111/ddi.12854
Retzlaff & Robrecht(2011)	Lepidoptera	Retzlaff, H., & Robrecht, D. (2011). Die Veränderung der Fauna der tagaktiven Großschmetterlinge im Kreis Lippe im Lauf der vergangenen 140 Jahre. Melanargia, 24, 193–268.
Scheffler(2017)	Carabidae	Scheffler, I. (2017). Bestandsveränderungen von Laufkäfern (Coleoptera, Carabidae) in Potsdam. Veröffentlichungen des Naturkundemuseums Potsdam, 3, 45–58.
Schiess & Schiess-Bühler(1997)	Lepidoptera	Schiess, H., & Schiess-Bühler, C. (1997). Dominanzminderung als ökologisches Prinzip: Eine Neubewertung der ursprünglichen Waldnutzungen für den Arten- und Biotopschutz am Beispiel der Tagfalterfauna eines Auenwaldes in der Nordschweiz. Mitt. Eidgenöss. Forsch.anst. Wald Schnee Landsch., 125.
Schröter & Irmler(2013)	Carabidae	Schröter, L., & Irmler, U. (2013). Organic cultivation reduces barrier effect of arable fields on species diversity. Agriculture, Ecosystems & Environment, 164, 176–180. https://doi.org/10.1016/j.agee.2012.09.018
Schwerk et al(2006)	Carabidae	Schwerk, A., Duszczyk, M., Sałek, P., Abs, M., & Szyszko, J. (2006). Variability of Carabidae in time and space in open areas. Entomologica Fennica, 17(3), 258–268. https://doi.org/10.33338/ef.84341
Shortall et al(2009)	Lepidoptera	Shortall, C. R., Moore, A., Smith, E., Hall, M. J., Woiwod, I. P., & Harrington, R. (2009). Long-term changes in the abundance of flying insects. Insect Conservation and Diversity, 2(4), 251–260. https://doi.org/10.1111/j.1752-4598.2009.00062.x
Taylor & Morecroft(2009)	Lepidoptera, Carabidae	Taylor, M. E., & Morecroft, M. D. (2009). Effects of agri-environment schemes in a long-term ecological time series. Agriculture, Ecosystems & Environment, 130(1–2), 9–15. https://doi.org/10.1016/j.agee.2008.11.004
Thomas et al(2004)	Lepidoptera	Thomas, J. A., Telfer, M. G., Roy, D. B., Preston, C. D., Greenwood, J. J. D., Asher, J., Fox, R., Clarke, R. T., & Lawton, J. H. (2004). Comparative Losses of British Butterflies, Birds, and Plants and the Global Extinction Crisis. Science, 303(5665), 1879–1881. https://doi.org/10.1126/science.1095046
Turin & Den Boer(1988)	Carabidae	Turin, H., & Den Boer, P. J. (1988). Changes in the distribution of carabid beetles in The Netherlands since 1880. II. Isolation of habitats and long-term time trends in the occurence of carabid species with different powers of dispersal (Coleoptera, Carabidae). Biological Conservation, 44(3), 179–200.
Van Dijk(1987)	Carabidae	Van Dijk, T. S. (1987). The long-term effects on the carabid fauna of nutrient impoverishment of a previously arable field. Acta Phytopathologica et Entomologica Hungarica, 22(1–4), 103–118.
Van Dyck et al(2009)	Lepidoptera	Van Dyck, H., Van Strien, A. J., Maes, D., & Van Swaay, C. A. M. (2009). Declines in Common, Widespread Butterflies in a Landscape under Intense Human Use. Conservation Biology, 23(4), 957–965. https://doi.org/10.1111/j.1523-1739.2009.01175.x
Van Langeveld et al(2018)	Lepidoptera	Langevelde, F., Braamburg-Annegarn, M., Huigens, M. E., Groendijk, R., Poitevin, O., Deijk, J. R., Ellis, W. N., Grunsven, R. H. A., Vos, R., Vos, R. A., Franzén, M., & WallisDeVries, M. F. (2018). Declines in moth populations stress the need for conserving dark nights. Global Change Biology, 24(3), 925–932. https://doi.org/10.1111/gcb.14008
Van Noordwijk et al(2017)	Carabidae	van Noordwijk, C. G. E., Baeten, L., Turin, H., Heijerman, T., Alders, K., Boer, P., Mabelis, A. A., Aukema, B., Noordam, A., Remke, E., Siepel, H., Berg, M. P., & Bonte, D. (2017). 17 years of grassland management leads to parallel local and regional biodiversity shifts among a wide range of taxonomic groups. Biodiversity and Conservation, 26(3), 717–734. https://doi.org/10.1007/s10531-016-1269-5

Van Strien et al(2019)	Lepidoptera	Van Strien, A. J., Van Swaay, C. A. M., van Strien-van Liempt, W. T. F. H., Poot, M. J. M., & WallisDeVries, M. F. (2019). Over a century of data reveal more than 80% decline in butterflies in the Netherlands. Biological Conservation, 234, 116–122. https://doi.org/10.1016/j.biocon.2019.03.023
Van Swaay et al(2018)	Lepidoptera	Van Swaay, C. A. M., Bos, G., van Grunsven, R. H. A., Kok, J., Huskens, K., van Deijk, J. R., & Poot, M. (2018). Vlinders en libellen geteld. Jaarverslag 2017. Rapport VS2018. 006 [Butterflies and Dragonflies Counted. Annual Report 2017. Report S2018. 006].
Van Swaay(1990)	Lepidoptera	Van Swaay, C. A. M. (1990). An assessment of the changes in butterfly abundance in The Netherlands during the 20th Century. Biological Conservation, 52(4), 287–302. https://doi.org/10.1016/0006-3207(90)90073-X
WallisDeVries & Van Swaay(2006)	Lepidoptera	WallisDeVries, M. F., & Van Swaay, C. A. M. (2006). Global warming and excess nitrogen may induce butterfly decline by microclimatic cooling. Global Change Biology, 12(9), 1620–1626. https://doi.org/10.1111/j.1365-2486.2006.01202.x
WallisDeVries & Van Swaay(2016)	Lepidoptera	WallisDeVries, M. F., & Van Swaay, C. A. M. (2017). A nitrogen index to track changes in butterfly species assemblages under nitrogen deposition. Biological Conservation, 212, 448–453. https://doi.org/10.1016/j.biocon.2016.11.029
WallisDeVries et al(2012)	Lepidoptera	WallisDeVries, M. F., Van Swaay, C. A. M., & Plate, C. L. (2012). Changes in nectar supply: A possible cause of widespread butterfly decline. Current Zoology, 58(3), 384–391. https://doi.org/10.1093/czoolo/58.3.384
Warren et al(2001)	Lepidoptera	Warren, M. S., Hill, J. K., Thomas, J. A., Asher, J., Fox, R., Huntley, B., Roy, D. B., Willis, S. G., & Greatorex-Davies, J. N. (2001). Rapid responses of British butter¯ies to opposing forces of climate and habitat change. 414, 5.
Wenzel et al(2005)	Lepidoptera	Wenzel, M., Schmitt, T., Weitzel, M., & Seitz, A. (2006). The severe decline of butterflies on western German calcareous grasslands during the last 30 years: A conservation problem. Biological Conservation, 128(4), 542–552. https://doi.org/10.1016/j.biocon.2005.10.022
Woiwod & Harrington(1994)	Lepidoptera	Woiwod, I. P., & Harrington, R. (1994). Flying in the face of change: The Rothamsted Insect Survey. Long-Term Experiments in Agricultural and Ecological Sciences, 321–342.
Wurth(2004)	Carabidae	Wurth, C. (2004). Auswirkungen einer 13jährigen extensiven Beweidung auf die Laufkäferfauna von pannonischen Trockenrasen im Naturschutzgebiet 'Hundsheimer Berge' (Niederösterreich). Angewandte Carabidologie Supplement, 3, 59−66.
