# Supplementary material for: Drivers and pressures behind insect decline in Central and Western Europe based on long-term monitoring data
Source: PLoS One. 2023 Aug 23;18(8):e0289565. doi: 10.1371/journal.pone.0289565 (PMC10446172; doi:10.1371/journal.pone.0289565)

Figure S1: Analysed publications and their recording timespan and recording intervals, respectively. (a) overview of publications per country in the focus region, (b) 33 publications with Carabidae trends, (c) 54 publications with Lepidoptera trends.

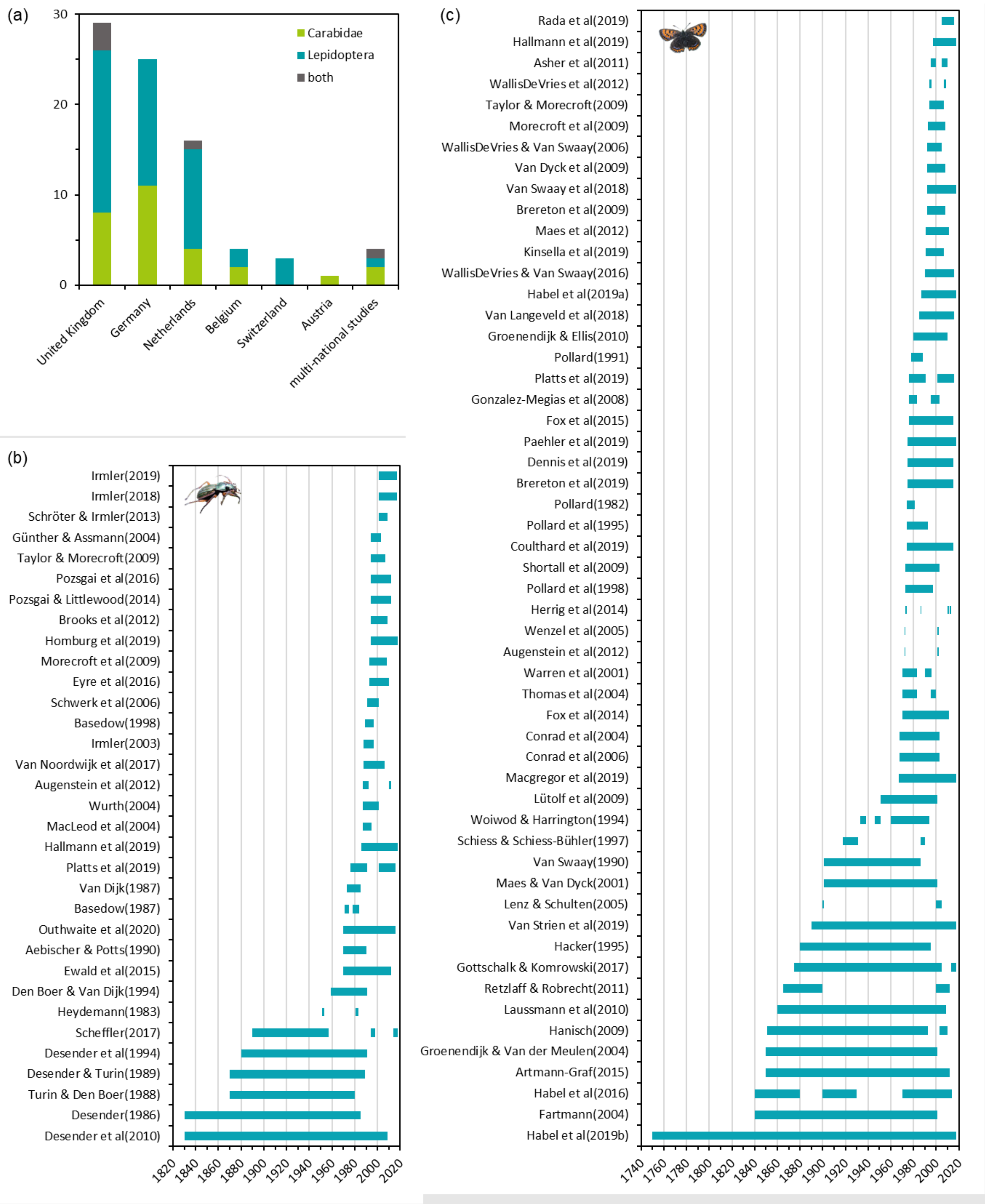

Supplement: S1 Fig — (a) overview of publications per country in the focus region, (b) 33 publications with Carabidae trends, (c) 54 publications with Lepidoptera trends. (PDF) [file pone.0289565.s006.pdf]
